# Observe, Digest, Concentrate, Act

**Published:** 1894-10

**Authors:** 


### Editorial, Etc.

OBSERVE, DIGEST, CONCENTRATE, ACT.—Triflers with diamonds under their feet, see only useless sand; spendthrifts, with handsful of pearls, think only of vanities; lazy men, with golden opportunities within reach, accomplish nothing. Oh, so many are interested with gew-gaws, while a world is offered them; they reflect on nonsense, while the stupendous problems of life are proposed to them; they trifle with a thousand bubbles, when one sensible pursuit would bring them riches, and honor, and glory. How they while away their precious time lazily when action would give them substantial good.

Talk about this world being in confusion and ruin; there is nothing wrong about man. If we trample on evil, and ob serve the good; shut our eyes on man's follies, and reflect on God's wonders; dismiss vageries, and seek true riches; if we cease busying ourselves with vanities, and become filled with good deeds, then this world within us and without us is a delectable field of transcendent sublimity, from which we may draw, and concentrate, and digest power to become what we will, and accomplish what we will, till we burst this cumbrous clay, and soar away to higher spheres.

But stop. Don't fly yet. Right where you are, and as you are, do what you can with what you have, and what surrounds you; this will make you what you would be. You cannot become what you may be by mere impulse, nor by some great leap, and you have no wings. Men grow; they do not fly till they are transformed into angles. They come to their best attainments here step by step—carefully observing, thoroughly digesting, wisely concentrating on the most important, and then deliberately acting with enthusiasm and intelligence. Thus we gather the crudities abcut us, and convert them into priceless values, and by using them wisely we may attain something approaching our ideal of life.

What business man succeeds without this power to wisely observe, digest, concentrate, and act? With these qualities few fail. This secret charm and strength is a talisman of prosperty. These four spirits of diligence strew on our path the aroma of health, the gold of wealth, and the precious gems of happiness. Honor and wisdom and triumph follow in their steps.

Teach a young man how to observe, and how to digest what he observes, and how to make use of what he digests; teach him how to make this his life habit, and you may leave him to himself. They will be sure to make him something worthy of his being. They are better than money—yes, than a whole mine of gold. He will be sure to create something from nothing that will improve the world, and in improving the world he will improve himself, till head and heart are centers of the strength and perfection of life. He has a key that opens every door, and makes him welcome wherever he wills to go. The hidden recesses of nature become familiar to him; the intricate problems of life are solved, and the most delightful associations are his home.

Christ says the heart of the pure is such a clear, beautiful, well shapen lens that the possessor can look through it and see God. So here we have a compound lens through which the possessor can see the glory of the world and possess it. It gives us eyes to see, powers to appropriate, skill to manipulate, and wisdom to act as monarch of all we survey. Having this, we can see flying all about us glorious possibilities—embrio worlds for our development, wonderful possessions that bring with them the power to transmute misfortune into fortune, dire defeats into triumphant feats, and poverty into riches.—Editorial in *Items of Interest.*

---

